# *Mimosa pudica* mucilage nanoparticles of losartan potassium: Characterization and pharmacodynamics evaluation

**DOI:** 10.1016/j.jsps.2023.101695

**Published:** 2023-07-06

**Authors:** Shazia Akram Ghumman, Arshad Mahmood, Sobia Noreen, Huma Hameed, Rizwana Kausar, Maria Rana, Asma Aslam

**Affiliations:** a College of Pharmacy, University of Sargodha, Sargodha 40100, Pakistan; b College of Pharmacy, Al Ain University, Abu Dhabi Campus, Abu Dhabi, UAE; c AAU Health and Biomedical Research Centre (HBRC), Al Ain University, UAE; d Institute of Chemistry, University of Sargodha, Sargodha 40100, Pakistan; e Faculty of Pharmaceutical Sciences, University of Central Punjab, Lahore 54000, Pakistan; f ILM College of Pharmaceutical Sciences, Sargodha 40100, Pakistan; g Riphah Institute of Pharmaceutical Sciences, Riphah International University Lahore Campus, Lahore 54000, Pakistan

**Keywords:** *Mimosa pudica mucilage*, Nanoparticles, Losartan potassium, Sustained effect

## Abstract

The current research was to develop nanoparticles based on *Mimosa pudica* mucilage (MPM) that could encapsulate losartan potassium (LP). Nanoparticles (NPs) produced through ionic-gelation method; the polymerization of the mucilage carried out using calcium chloride as cross-linking agent. The MPMLP-NPs demonstrated vastly enhanced pharmaceutical characteristics, presented discrete surface with spherical shape of 198.4–264.6 nm with PDI ranging 0.326–0.461 and entrapment efficiency was in the range of 80.65 ± 0.82–90.79 ± 0.96%. FTIR and DSC indicated the stability of drug during the formulation of nanoparticles. An acute oral toxicity investigation found no significant alterations in behavior and histopathology criteria. The MPMLP-NPs formulation revealed the better rates and sustained effect as assessed with the commercial product. Moreover, low dose of MPMLP-NPs showed similar anti-hypertensive effect as assessed with the marketed tablet.

## 1 Introduction

Hypertension is intimately correlated to cardiac and hepatic disorders, resulting in the deaths of nearly 10 million individuals and the impairment of above 200 million people (Zafarullah et al., 2015). Despite this, change in lifestyle can help manage the medical problem, however the importance of pharmacological therapy cannot be overstated (Williams et al., 2018). Angiotensin receptor blockers (ARB), angiotensin converting enzyme (ACE) inhibitors, calcium channel blockers and diuretics have been the most widely used treatments for hypertension. Losartan Potassium (LP), an orally available non-peptide member to the ARB class, is one of several drugs in this category. The LP as well as its active metabolite E 3174 are powerful AT-I receptor subtype inhibitors. Aside from it, if you stop using the medicine, you won't have any withdrawal symptoms (Goldberg et al., 1995). In spite of various characteristics, the bioavailability of LP is only 33%. It undergoes rapid conversion by CYP-450 enzyme, with 14% metabolized to its active metabolite. The LP and its active metabolite have half-lives of 2 h and 6–9 h, respectively (Delmarre et al., 2007, Rao et al., 2013). The short half-life problem, as well as side symptoms such as diarrhea, muscle cramps, vertigo, sleeplessness, rhinitis, dry cough, elevated blood potassium, and libido has necessitated the development of an alternate dosage form; whether reduce the frequency of dose or improve the drug's efficacy (Mokale et al., 2014). Strategies such as prolonged release formulations of lowering the drug's hepatic metabolism might be useful in increasing the efficiency of a medicine using smaller amounts (Kommana et al., 2020, Hameed et al., 2021).

Nanotechnology has established significant consideration in the last two decades from industries like chemistry, nano electronics, biosensor, biosciences, and pharmaceutical industries (Maeda et al., 2009, Capretto et al., 2013). The size and morphology of nanoparticles play a vital role in pharmaceutical product delivery such as prolonged release, controlled release, and the pharmacokinetics of removal from the body in the pharmaceutical industry (Appalakutti et al., 2015). Because of their physicochemical features and improved process control, nanoparticles have significant characteristics (Noreen et al., 2022). Controlled drug delivery, a significant quantity of drug absorption, and an enormous quantity of drug uptake by target tissues are all advantages of nanoparticles (Paques 2015).

The majority of research has focused on polymeric nanoparticles generated from biopolymers. Literature based research clears that natural polymers have many advantages over synthetic polymers (Hameed et al., 2015). Polysaccharides have a high rate of absorption and degradation, allowing for a significant cellular release (Noureen et al., 2022). Moreover, hydrophilic groups in its structural framework, such as hydroxyl, carboxyl, and amino groups, improve adhesion properties with endothelium and mucosa that is an effective technique for increasing drug bioavailability in targeted drug delivery (Naji-Tabasi et al., 2017). As a result, polysaccharides and natural products have a great potential as biopolymers for nanoparticle preparation, and identifying novel sources of biopolymers, particularly plant-derived polymers with desirable characteristics, is a major area of research (Ghumman et al., 2017).

Previously the capabilities of *Mimosa pudica* seed mucilage for sustained release examined (Singh et al., 2009). Considering its excellent safety characteristics, simplicity of accessibility and cheap price, *Mimosa pudica* mucilage is widely employed in food processing industries as additional ingredients. In pharmaceutical industry, MPM received a lot of attention as an emulsifying agents, stabilizers, and gel formers, suspending agent and drug release modifier (Anroop et al., 2006, Singh et al., 2009, Saeedi et al., 2015). According to the previous scientific report, no research done on the potential of *Mimosa pudica* mucilage (MPM) as a potential polysaccharides resource for developing new nanoparticles. The ionic gelation approach used to make MPMLPNPs since it is one of a few organic solvent-free procedures and nanoparticles completely manufactured in water. This process has a number of benefits, such as easy techniques, a moderate preparation method that avoids any use of organic solvents, heating, or rapid stirring, all of which might harm sensitive compounds (Pedroso-Santana and Fleitas-Salazar, 2020). MPM generates bonds between its negatively charged group and tiny ions with oppositely charged, such as calcium, in inotropic gelation.

The current study objectives were to (i) investigate the effect of various ratios of MPM on the physicochemical properties; i.e.: particle size, yield and drug content of MPMLP-NPs;(ii) examine surface morphology through SEM (iii) characterize drug-polymer interaction and compatibility through the techniques like FTIR and DSC (iv) evaluate *in vitro* drug release profile at pH 7.4 (v) formulation based on good *in vitro* results were chosen for *in vivo* toxicity testing in a rat model out of three optimized MPMLP-NPs. Based on *in vitro* characterization and acute toxicity study, the nanoparticles formulation chosen for *in vivo* pharmacodynamics testing that was further compared with available marketed formulation both at *in vitro* and *in vivo* level to confirm the sustained effect of prepared formulations.

## 2 Experimental

### 2.1 Materials

Seeds of *Mimosa pudica* obtained from local market of Sargodha. Losartan potassium received from Wilson Pharmaceuticals (Pvt) Ltd. Sodium hydroxide, Dimethylsulphoxide (DMSO), Calcium chloride, Potassium dihydrogen phosphate (Merck). Ethanol supplied by Sigma-Aldrich. Analytical grade solvents employed throughout the research. Marketed formulation was (Qsartan of High-Q Pharmaceuticals Pvt. Ltd.).

### 2.2 Extraction of mucilage from *Mimosa pudica* seeds

For mucilage extraction, 50 g of *Mimosa pudica seeds* immersed for an h at 37 °C in distilled water. Seeds entirely moistened, swelled, and wrapped in a thick mucilage in an hour. Squeezing the entire blended combination of mucilage and seeds through the muslin fabric separated the aqueous mucilaginous extract. With the use of 96% ethanol, the polysaccharides of mucilage precipitated. Gradually, mucilage and ethanol blended in a 1:3 mucilage to ethanol ratio. The precipitated mucilage then collected and processed in glass plates in a hot air oven at 38 °C until the polymer was entirely dry.

### 2.3 Preparation of MPMLP-NPs

Various quantities of polymer (MPM) and cross-linking agents (CaCl_2_ and DMSO) was used to make losartan potassium nanoparticles as shown in Table 1. Solution A made by dispersing MPM in distilled water, 5 ml of 0.1 M NaOH was added to the polymer solution and then adding LP solution. For the preparation of solution B, CaCl_2_ dissolved in small quantity of water and further mixed with Dimethyl sulphoxide (DMSO) to form the cross-linking agent. Furthermore, solution B was gradually added to a solution A, using a syringe with constant magnetic stirring of 250 rpm, and the resulting suspension was sonicated at a pulse mode for 90 s. After that, the suspension centrifuged at 4000 rpm for 15 min at 37 °C. The supernatant discarded and pellet washed with water (2 × 10 ml) to remove DMSO. The sediments (nanoparticles) was left to dry in a hot air oven at 38 °C for 48 h and then stored (Venier-Julienne and Benoit, 1996, Petronijevic et al., 2013, Chopra et al., 2016, Naji-Tabasi et al., 2017, Wee et al., 2017).

**Table 1:** Composition of MPMLP-NPs formulations crosslinking with (CaCl_2_ + DMSO).

Formulations	LP (mg)	MPM	NaOH (ml)	CaCl_2_ (g)	DMSO (ml)
F1	150	0.25	5	3	50
F2	150	0.25	5	5	70
F3	150	0.25	5	7	100
F4	150	0.5	5	3	50
F5	150	0.5	5	5	70
F6	150	0.5	5	7	100
F7	150	1	5	3	50
F8	150	1	5	5	70
F9	150	1	5	7	100

LP, Losartan Potassium. MPM, *Mimosa pudica* mucilage. NaOH, Sodium hydroxide. CaCl_2_, Calcium chloride. DMSO, Dimethyl sulphoxide.

### 2.4 Fourier-transform infrared (FTIR) spectroscopy

The FTIR approach was used to investigate the compatibility of drug and polymers used, allowing the functional groups of the polymer, drug, and their interactions to be detected. It also aids in the investigation of bond vibrations inside compounds. To make the tablet, the dried nanoparticles were ground into a fine powder and combined with KBr. The FTIR spectra was scanned at a range of 4000 to 400 cm^−1^ (El-Gizawy et al., 2019).

### 2.5 Differential scanning calorimetry (DSC)

DSC analysis performed for thermodynamic analysis of the MPM, LP and MPMLP-NPsIn the aluminum pan sealed with the perforated aluminum cover, the sample fed into the DSC equipment (TA Instruments Trios V4.1). The scanning temperature maintained at a rate of 10 °C/min for 50–300 °C. Nitrogen gas was passed 50 ml/min to keep the environment inert (Antoniraj et al., 2020).

### 2.6 Drug content and percentage yield

To test drug content centrifugation method was used. The suspension centrifuged at 15000 rpm for 40 min at 25 °C to extract the free drug. The supernatent collected from the free drug and purified via whatman filter paper. The absorbance in a UV spectrophotometer at 206 nm was then used to calculate the% drug content (Koo et al., 2011, Singh, 2011). The % yield was determined as described below, the weight of drug and polymer along with weight of nanoparticles recovered.

$$\%\;Drug\;Content\;=\frac{Weight\;of\;drug\;in\;nanoparticles}{Weight\;of\;nanoparticles\;recovered}\times 100$$

$$\%\;Yield=\frac{Weight\;of\;nanoparticles\;recovered}{Weight\;of\;total\;solid\;(MSM+LP)}\times 100$$

### 2.7 Morphology of MPMLP-NPs

MPMLP-NPs subjected to SEM analysis on scanning electron microscope (JEOL 1.1, JSM-5910). Samples mounted on the holder and layered with the gold film to provide even conductivity surface and smooth analysis. System was operated under vacuum at different magnifications (Vladár and Hodoroaba 2020).

### 2.8 Zeta size and zeta potential of MPMLP-NPs

The particle size, polydispersity index (PDI), and zeta potential of MPMLP-NPs were evaluated using Malvern Instruments Nano ZS 90 Zeta-sizer (Malvern, UK). To eliminate repetitive scattering, a He/Ne laser and water as a dispersion used (Refractive index: 1.33). Zeta potential and polydispersity index were also determined. Concentrated solution may lead to false results of particle size analysis due to multiple scattering. Therefore, 1 mg/mL of MPMLP-NPs diluted solution used for analysis. Data analyzed by cumulant method of analysis in Malvern software. This software considered each particle as a sphere and considered that in bulk distribution. To avoid particle aggregation tendency, samples added by using a 0.2 mm syringe filter. Collected results averaged of thrice analysis of each formulation. Data obtained also used for polydispersity index (PDI) analysis. (Naji-Tabasi et al., 2017). Polydispersity index that will give some idea about the degree of the particle size distribution of nanoparticles. Polydispersity index is determined using the following formula:

$$PDI=\frac{\mathrm{Mw}}{\mathrm{Mn}}$$

*Mw* indicates the average weight while *Mn* is representative of average number.

### 2.9 *In vitro* drug release studies

The process of dissolution performed on the dissolution apparatus USP Type II (Pharma max. TEST), at pH 7.4. Precisely weighed samples were placed in the diffusion dialysis bag and that was suspended in the 250 ml dissolution medium at 37 °C and constant stirring by 100 rpm at 1.2 pH for 2 h. Afterwards the dissolution media was replaced with phosphate buffer pH 7.4 for upto 24 h. Samples taken out from the dissolution medium with the immediate replacement of the equal quantity of media. Extracted samples were then set to UV spectrophotometer in order to observe the absorbance values using phosphate buffer pH 7.4 as a blank at λ_max_ 206 nm (Khan et al., 2022a, Khan et al., 2022b).

### 2.10 Drug release kinetics

Entire data of cumulative release of the drug applied to various kinetic models of data treatment i.e. Zero-Order, First-Order, Higuchi model, Korsmeyer-Peppas model and Hixson-Crowell’s model of kinetics. The best fitted model with the appropriate regression coefficient values was selected for the formulations (Khairnar et al., 2017).

### 2.11 *In-vivo* acute toxicity study of MPMLP-NPs

Prepared formulations also tested for acute toxicity of MP mucilage. Tests carried out by repeated dose technique over a period of 14 days following OECD guidelines. To carry out the tests approval taken from the ethical committee of the University of Sargodha, Sargodha, Pakistan (Ref. No 84A23 IAEC/UOS/PREC). For this, Adult male Wister albino rats weighing between 250 and 300 g purchased from university of Sargodha (UOS) animal facility. All the rats placed in a clean house facility with free access of food and water. Rats were equally dispersed into two groups (n = 8); one group as control and other group as tested one. Both groups maintained with free access of water and lab diet and second group was treated with MPMLP-NPs at the dose of 10 mg/kg once daily for 14 days. Daily basis observation carried out to see the signs of any illness, side effects or any sort of changes in the vital signs over the period of 2 weeks. The body weights of all animals also assessed before and after the treatment on daily basis upto 2 weeks. At the end, all readings compared with the control group. Biochemical and hematological profiling linked results also noted. All the animals euthanized and blood samples collected via orbital sinus route. All the blood samples were analyzed according to the IFCC guidelines as primary reference procedures using a chemistry analyzer® (Olympus AU2700 Optical, Tokyo, Japan) (Noreen et al., 2022).

### 2.12 Pharmacodynamics study

To carry out this test, 30 male adult Wister rats weighed between 190 and 200 g used. These rats randomly divided into 5 groups containing 6 rats each, they kept at 37 °C, with relative humidity 40–45% and 12-hour light/dark rotation. All rats had easy accessibility towards the food. The effectiveness of the F6 formulation evaluated using the fructose-induced hypertension model. S1 = fed with just bottled water; whereas S2 = diseased (10% Fructose solution) with no drug treatment, S3 = diseased (10% w/v fructose solution) + blank formulation, S4 = diseased (10% w/v fructose solution) + MF and S5 = diseased (10% w/v fructose solution) + F6. The blood pressure regularly checked for days 0, 1,3,5,7,9,12 and 14th day. The rat dose was determined by using the following formula listed below (Nair and Jacob 2016). The blood pressure (Systolic) was measured at different times 0, 1, 3, 5, 7, 9, 12 and 14th day by using tail cuff method using noninvasive blood pressure recorder (Ugo Basile) before and after the given treatment. This method is quite accurate for measuring blood pressure in awake Wister rats. Concisely, the tail placed inside a cuff that was elongate, expanded and had a piezoelectric pulse sensor at its posterior portion. Once the initial wave occurred upon that sensor, the cuffs pressure being slowly reduced, and blood pressure being taken (Maiti et al., 2007).

$$Rat\;dose\;\left(\frac{mg}{kg}\right)\times 0.16=Human\;dose\;\left(mg/kg\right).$$

## 3 Results

The extracted MPM evaluated for physicochemical properties after it had fully dried. The physicochemical properties of extracted MPM showed color off-white, odorless, and tasteless. It was soluble in hot water and swelled in cold water. MPMLP-NPs prepared by mixing of two solutions (A&B); NaOH added in solution A act as a stabilizing agent; DMSO was added to CaCl_2_ crosslinking solution B to improve the cross-linking reaction. Following tests performed for the characterization of MPMLP-NPs.

### 3.1 Fourier-Transform Infrared (FTIR) spectroscopy

**Fig. 1:**
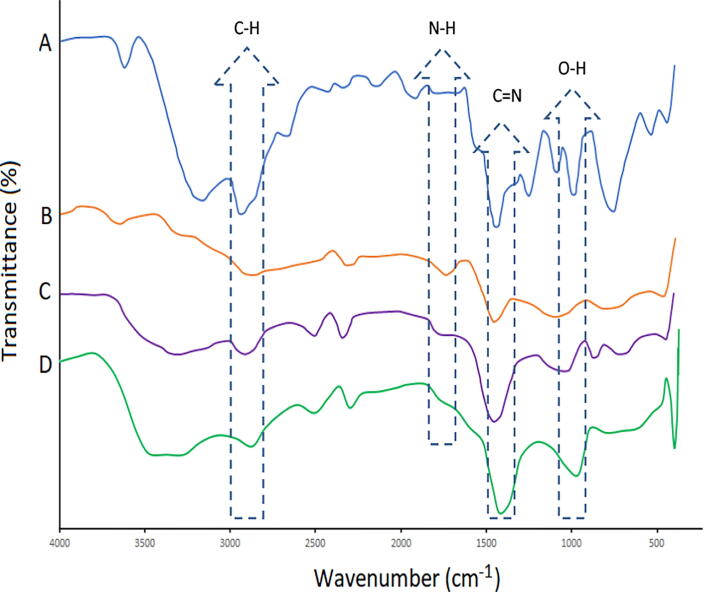
FTIR Spectra of (A) LP; (B) MPM; (C) MPMLP-NPs; (D) Unloaded nanoparticles.

FTIR spectra of LP, MPM, loaded MPMLP-NPs and unloaded MPMLP-NPs are shown in Fig. 1. FTIR spectrum of MPM represented distinguishable bands around 1165.0–1741.71 cm^−1^, the band around 1165.0–1259.0 cm^−1^ showed the existence of carbohydrates, whereas bands near 1350.17 cm^−1^ and 1448.54 cm^−1^ confirmed the presence of aromatics (Mumtaz et al., 2019).

The IR spectra of LP revealed a wide absorption group at 3197.9 cm^−1^ and dual groups at 995.27 and 1008.7 cm^−1^ attributable to tetrazole band and a strong peak at 1458.18 cm^−1^ related to imidazole ring. The distinctive peaks of O—H and C—O stretch appeared at 933.5 and 1072.4 cm^−1^. N—H stretch represented a group at 2954 cm^−1^ and C=N stretch provides one more one at 1423.47 cm^−1^ and triple amine group (C—N stretch) at 1260 cm^−1^ (Amer et al., 2019).

### 3.2 Differential Scanning Calorimetry (DSC)

**Fig. 2:**
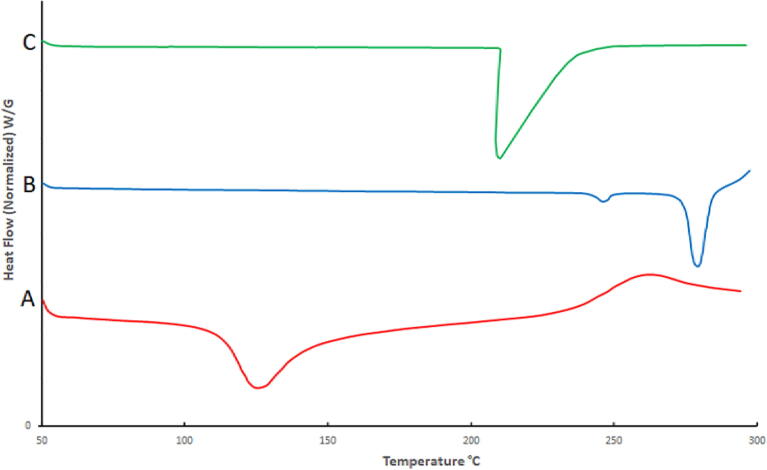
DSC of (A) MPM; (B) LP; (C) MPMLP-NPs.

The thermal analysis of *Mimosa pudica* mucilage Fig. 2(A) revealed a wide endotherm at 129 °C with the fusion energy of 116.1 J/g, demonstrating the amorphous structure of MPM (Ahuja et al., 2011). Thermograph of the pure drug LP Fig. 2(B) displayed a distinct sharp endothermic peak around 269.39 °C represented its melting point (El-Arabya et al., 2020). Furthermore, the thermogram of MPMLP-NPs, Fig. 2(C) demonstrated the distinct endotherm of the LP at 269.92 °C that intimated the crystalline nature of drug (Amer et al., 2019, El-Gizawy et al., 2019).

### 3.3 Drug content and percent yield

MPMLP-NPs were found to have drug content between 80.86 ± 0.92 and 90.79 ± 0.96%, while the % yield was found to be between 42.97 ± 0.74% and 84.89 ± 0.92% as shown in Table 2. The formulation F6 having 0.5 mg of MPM with cross-linking agent CaCl_2_ of 7% along with 100 ml of DMSO showed increased quantity of drug content and percentage yield.

**Table 2:** Characterization of MPMLP-NPs.

Formulations	% Drug Content	% Yield	Z-Average (nm)	PDI	Z-Potential (mV)
F1	82.29 ± 1.07	42.97 ± 0.74	234.9 nm	0.328	−14.6
F2	80.86 ± 0.92	56.98 ± 0.98	222.8 nm	0.436	−12.7
F3	82.74 ± 1.02	62.78 ± 1.08	264.6 nm	0.296	+11.2
F4	84.05 ± 0.74	64.55 ± 0.82	214.9 nm	0.376	−10.4
F5	80.65 ± 0.82	70.75 ± 0.94	278.2 nm	0.461	−12.4
F6	90.79 ± 0.96	84.89 ± 0.92	198.4 nm	0.363	−14.9
F7	84.75 ± 1.02	76.62 ± 1.07	257.9 nm	0.402	−11.8
F8	82.92 ± 0.82	75.29 ± 0.72	229.1 nm	0.426	−12.9
F9	88.72 ± 0.98	80.09 ± 1.08	204.8 nm	0.326	−16.7

### 3.4 Scanning Electron Microscopy (SEM)

**Fig. 3:**
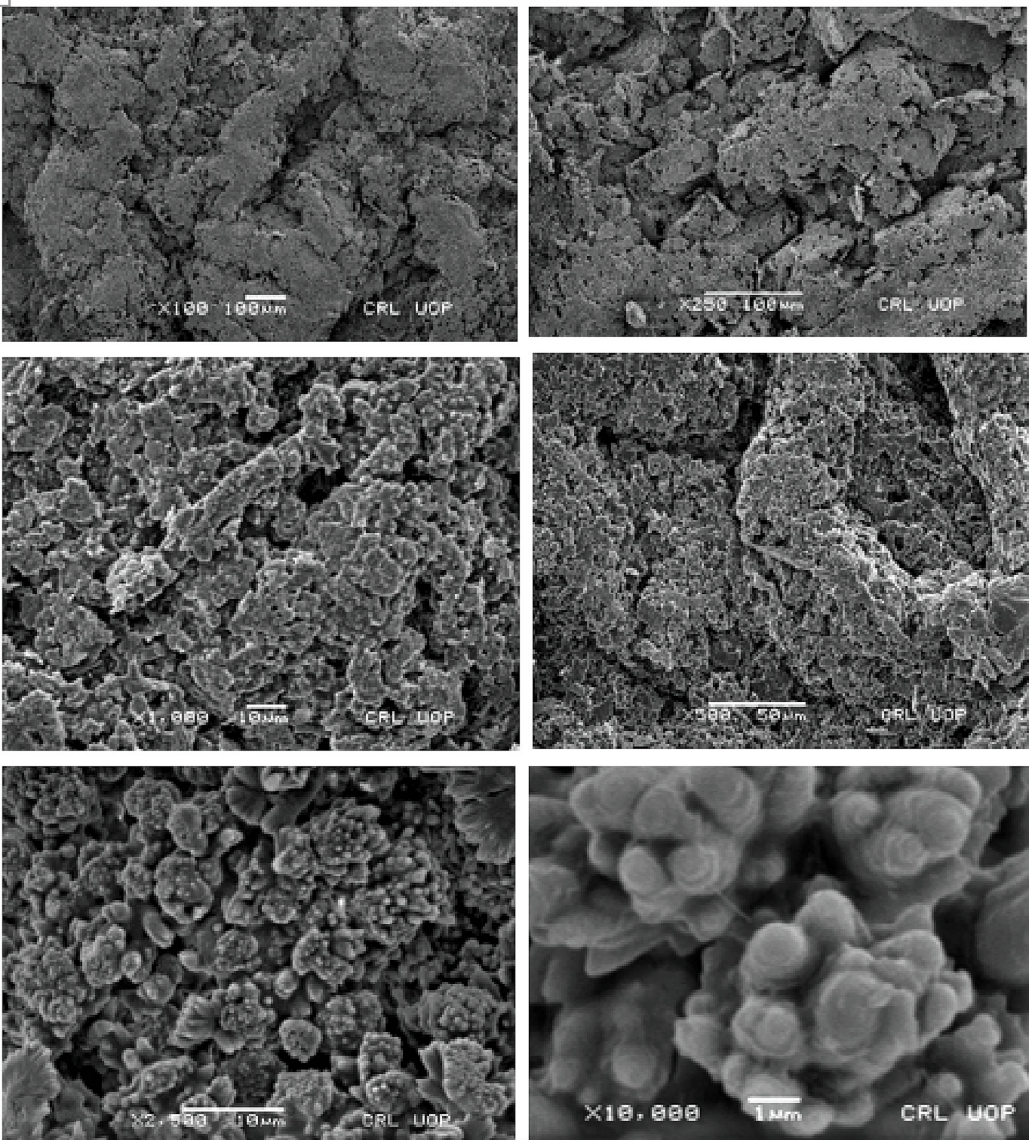
SEM images of optimized formulation F6.

SEM images revealed that the particles were of the similar size and the uniformity was observed which was also consistent with the value of PDI observed through zeta sizer and supported the stability of the nanoparticles. Moreover, the external surface of the particles was smooth and solid as shown in Fig. 3.

### 3.5 Particle size & zeta potential

**Fig. 4:**
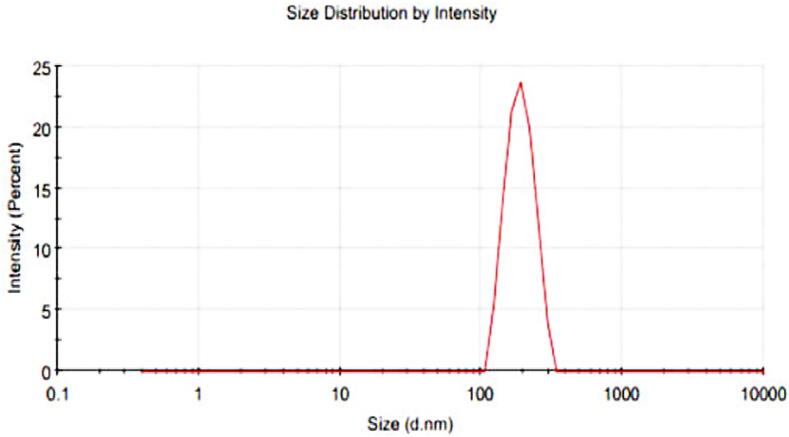
Zeta Size of the LP loaded MSM nanoparticles (F6).

**Fig. 5:**
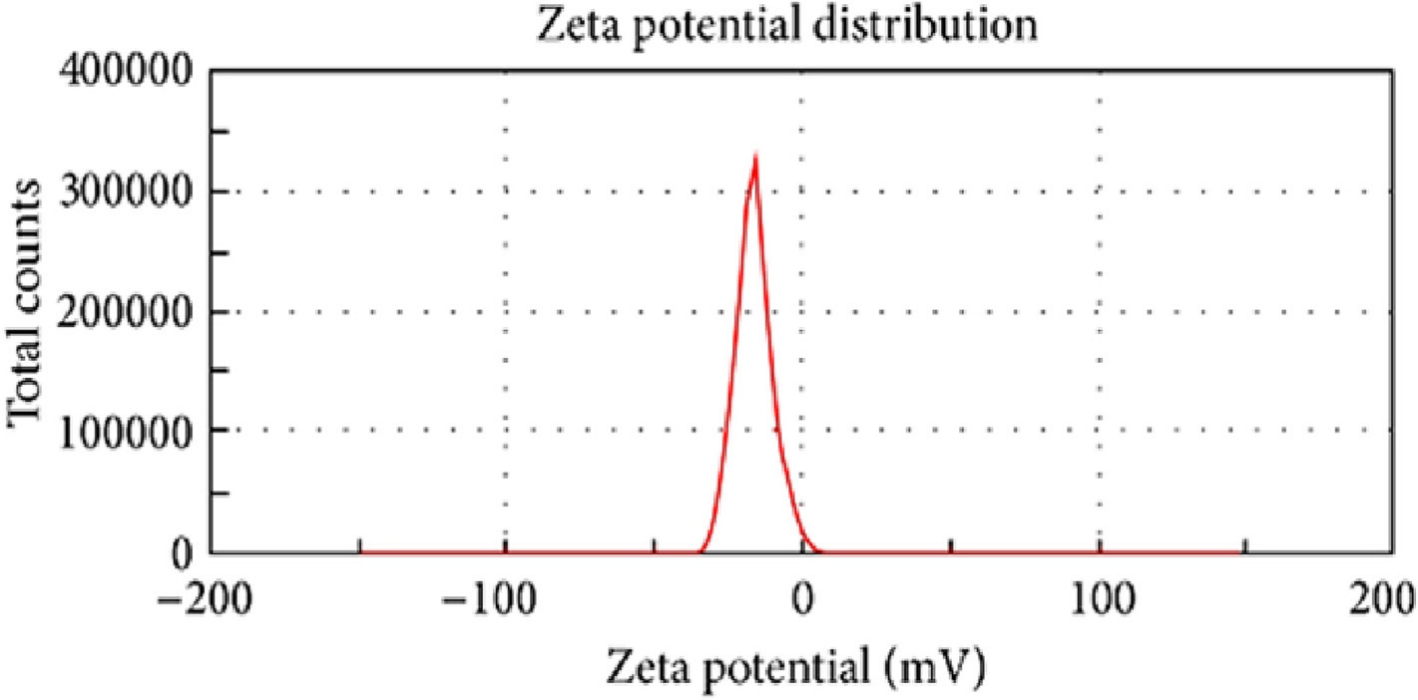
Zeta potential of MPMLP-NPs (F6).

The particle size, polydispersity index (PDI), and zeta potential of MPMLP-NPs were evaluated using Malvern Instruments Nano ZS 90 Zeta-sizer (Malvern, UK) as shown in Figs. 4 and 5. The graph indicated the single peak and peak was observed between 200 and 300 nm. Polydispersity index found within the range of 0.326–0.461. Zeta potential of the MPMLP-NPs was also determined through electrophoretic cell of the Malvern Instruments Nano ZS 90 Zeta-sizer and the charge on nanoparticles surface analyzed. F6 showed a single peak of zeta potential at −14.9 mV.

### 3.6 *In vitro* drug release studies

**Fig. 6:**
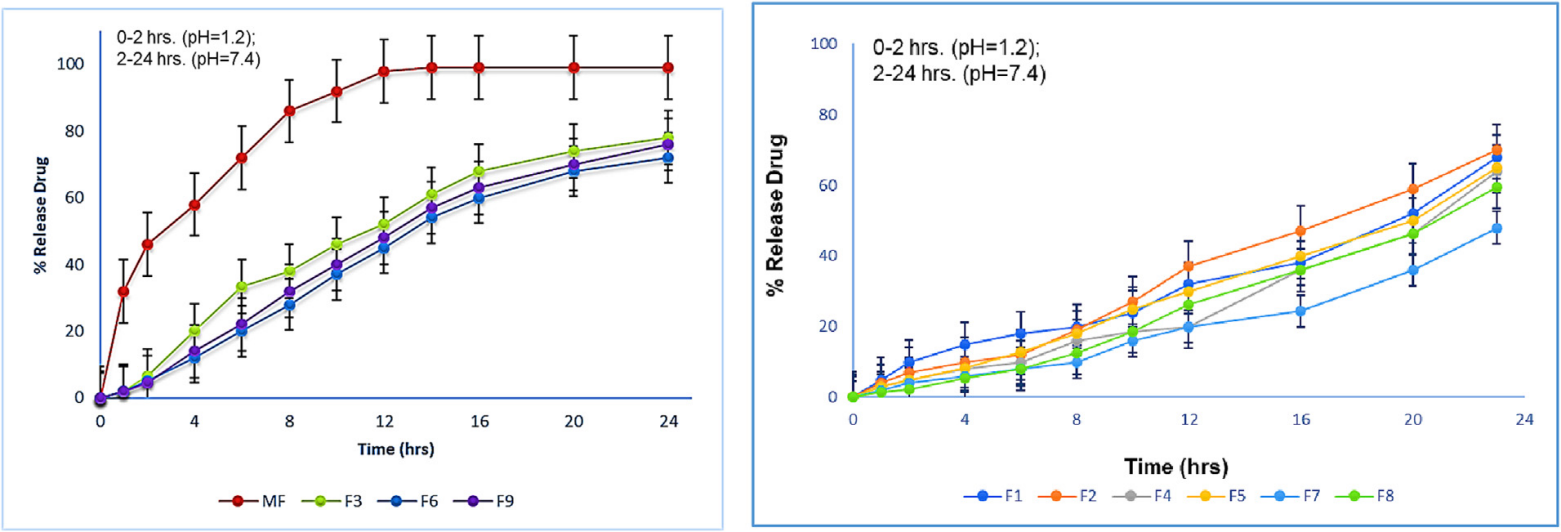
*In vitro* drug release profile of MPMLP-NPs F1-F9 and MF (Qsartan) at 37 °C in pH 1.2 for 2 h followed by pH 7.4 upto 24 h.

The *in-vitro* dissolution data was calculated for the prepared formulations (F3, F6 and F9) and one commercial product (Qsartan 25 mg). Dissolution samples were collected at the regular intervals upto 24 h and the percentage cumulative drug release profile for all the prepared and commercial formulation was established as shown in Fig. 6.

### 3.7 Drug release kinetic models

The results obtained from the entire *in-vitro* release studies studied through the basic kinetics models i.e. Zero-Order, First-Order, Higuchi, Korsmeyer-Peppas and Hixon-Crowell models. Table 3 summarized basic and important parameters of three formulations derived from the application of kinetic models.

**Table 3:** Results of kinetic model fitting of F3, F6 and F9.

Model	Parameter	F3	F6	F9
Zero-Order Model	R2 MSC	0.8271 1.5036	0.8783 1.8965	0.8623 1.7388
First-Order Model	R^2^ MSC	0.8305 1.5090	0.8737 1.7762	0.8852 1.8917
Higuchi Model	R^2^ MSC	0.6658 0.8303	0.7333 1.0281	0.6973 0.9221
Korsmeyer-Peppas Model	R^2^ MSC n	0.9940 3.807 1.292	0.9992 4.942 1.316	0.9927 3.0158 1.235
Hixson-Crowell Model	R^2^ MSC	0.8570 1.6795	0.9024 2.3307	0.8140 2.0567

### 3.8 In-vivo acute toxicity study of MPMLP-NPs

**Fig. 7:**
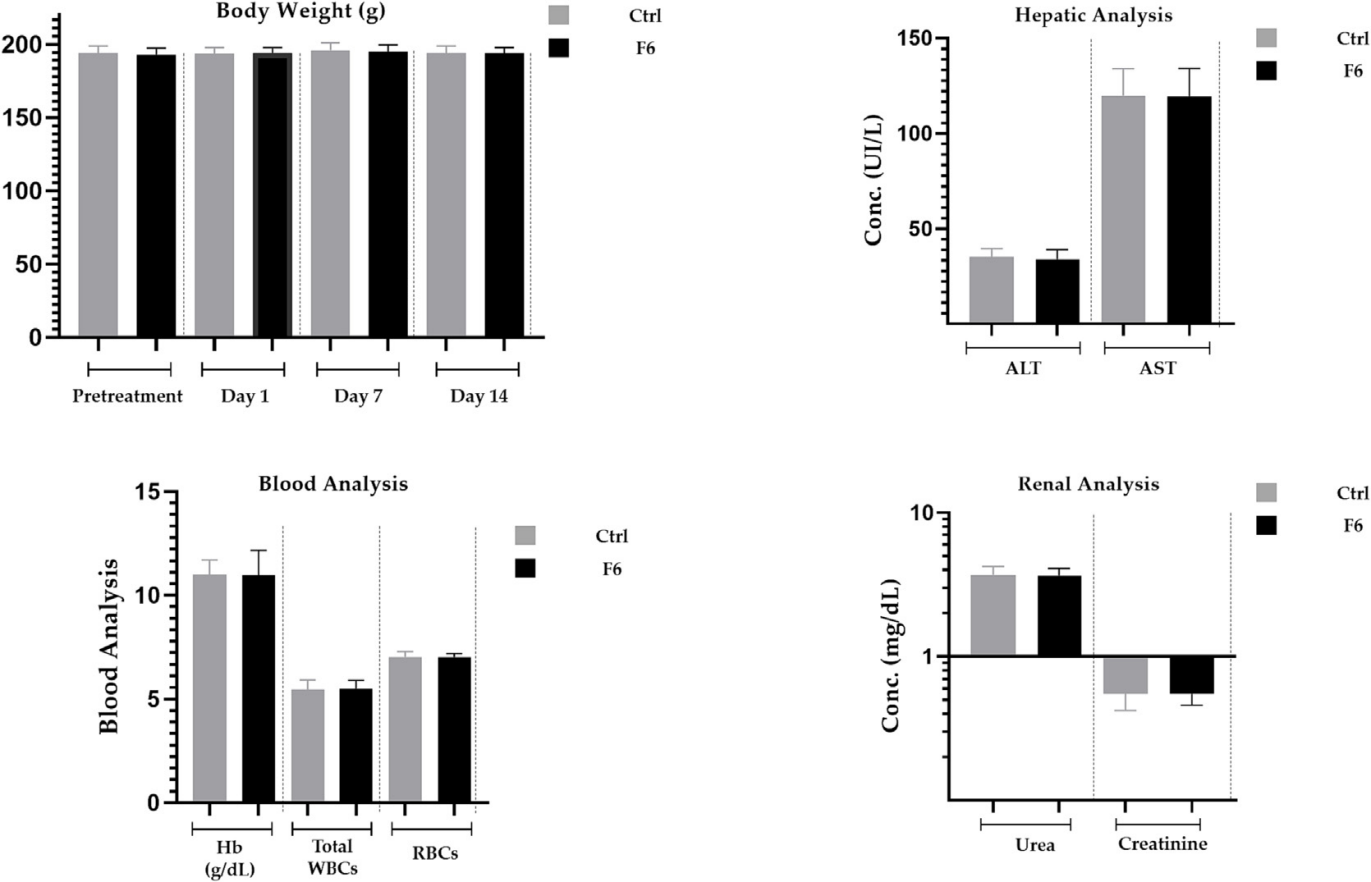
Biochemical analysis of Wistar Rats after repeated dose administration over a time, duration of 14 days; (a) body weight, (b) blood analysis, (c) hepatic analysis and (d) renal analysis Results were mentioned in as mean ± S.D. with n = 8.

**Fig. 8:**
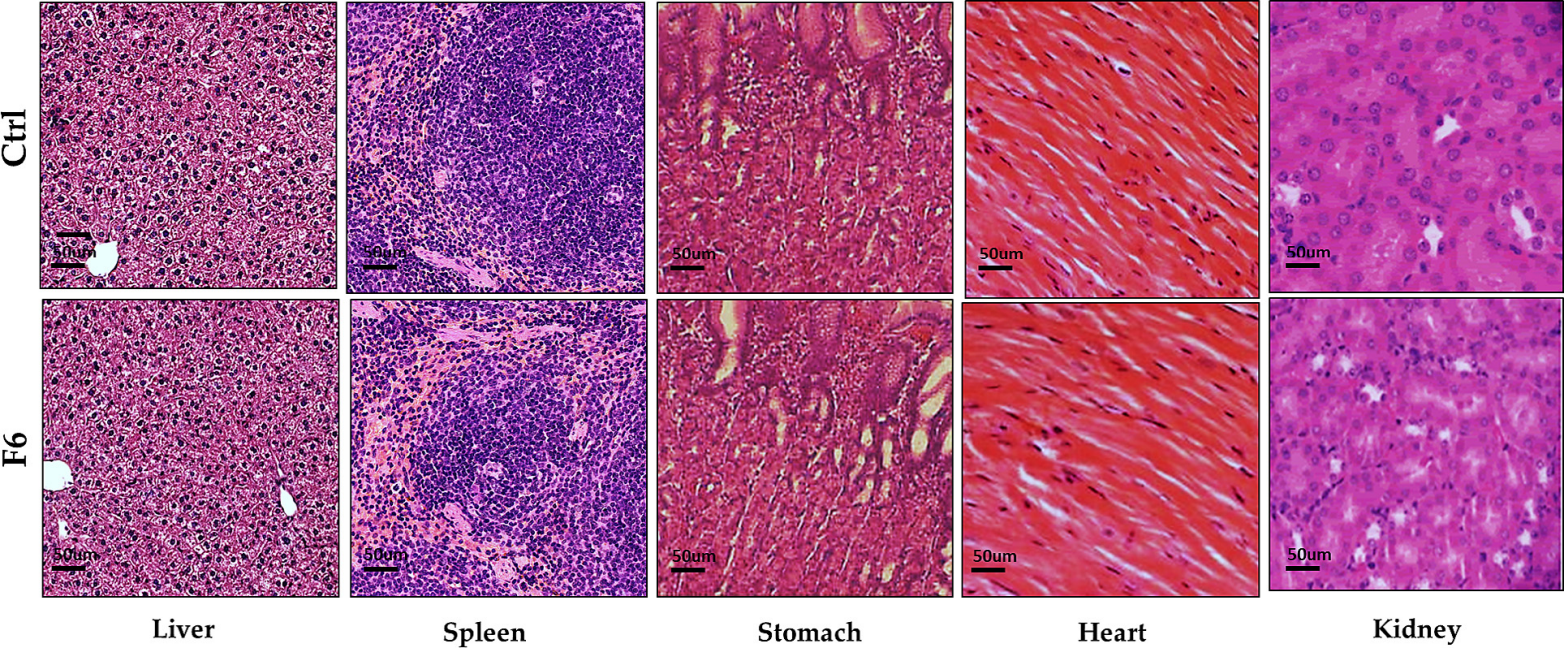
Histological analysis of vital organs in control and MPMLP-NPs treated groups.

Daily basis observation carried out to see the signs of any illness, side effects or any sort of changes in the vital signs over the period of 2 weeks. After carrying out this studyno sign of toxicity linked to newly designed formulation was appeared. Body weight, biochemical analysis and tissue histology data of vital organs mentioned in below Fig. 7, Fig. 8 and Table 4.

**Table 4:** Weight (g) of different vital organs in control and MPMLP-NPs treated groups.

Group	Liver	Stomach	Heart	Kidney	Spleen
Control	2.96 ± 1.02	1.47 ± 0.64	0.34 ± 0.04	0.39 ± 0.06	0.32 ± 0.01
Treated	3.06 ± 0.89	1.50 ± 0.34	0.32 ± 0.08	0.41 ± 0.04	0.29 ± 0.03

### 3.9 Pharmacodynamics analysis of MPMLP-NPs with marketed formulation (Qsartan)

**Fig. 9:**
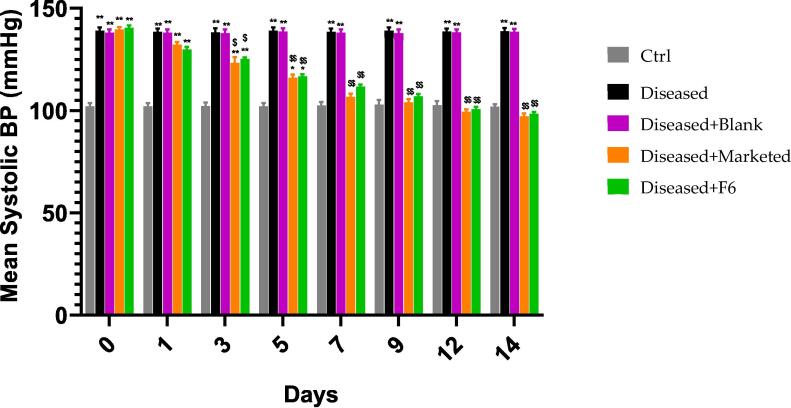
Mean systolic BP in different rat groups. S1 = Control (Received water only), S2 = diseased (10% Fructose solution) with no drug treatment, S3 = diseased (10% w/v fructose solution) + blank formulation, S4 = diseased (10% w/v fructose solution) + MF and S5 = diseased (10% w/v fructose solution) + F6. For all graphs, each column represents an individual group and errors bars are expressed as means ± SEM. (*p < 0.05; **p < 0.01; ***p < 0.001; * sign represents the difference of each group individually on comparison with the control group; ^$^ sign represents the difference of each group individually on comparison with the diseased group).

Following 14 days, S2, S3, S4, and S5 received a fructose mixture, freshwater, a control composition, a commercial pharmaceutical product, as well as the optimized preparation. The blood pressure (Systolic) was measured at different tome periods 0, 1, 3, 5, 7, 9, 12 and 14th day by using tail cuff method before and after the given treatment as mentioned in Table 5, Table 6 and Fig. 9.

**Table 5:** Rats group representation with Systolic blood pressure (mmHg) before drug treatment (10% w/v Fructose solution was given to S2 to S5 for the induction of hypertension).

Time (Days)	Systolic blood pressure (mmHg)				
	S1	S2	S3	S4	S5
0	104 ± 0.81	106 ± 0.75	105 ± 0.81	104 ± 0.56	103 ± 1.31
1	106 ± 0.81	106 ± 0.75	105 ± 0.81	105 ± 0.56	105 ± 2.52
3	104 ± 0.81	110 ± 0.56	111 ± 0.56	112 ± 1.23	116 ± 6.51
5	106 ± 6.64	112 ± 5.21	112 ± 1.23	125 ± 2.32	128 ± 3.54
7	105 ± 4.21	125 ± 6.21	116 ± 6.51	132 ± 5.64	134 ± 2.24
9	102 ± 4.42	132 ± 7.23	122 ± 4.12	138 ± 5.64	140 ± 2.24
12	102 ± 4.42	135 ± 7.23	136 ± 3.21	142 ± 1.23	143 ± 4.02
14	101 ± 2.12	137 ± 5.23	140 ± 3.12	146 ± 7.23	147 ± 3.33

**Table 6:** Rats group representation with Systolic blood pressure (mmHg) after treatment S1 = Control (Received water only), S2 = diseased (10% Fructose solution) with no drug treatment, S3 = diseased (10% w/v fructose solution) + blank formulation, S4 = diseased (10% w/v fructose solution) + MF (Qsartan) and S5 = diseased (10% w/v fructose solution) + F6.

Time (Days)	Systolic blood pressure (mmHg)				
	S1	S2	S3	S4	S5
0	101 ± 2.12	137 ± 5.23	140 ± 3.12	146 ± 7.23	147 ± 3.33
1	101 ± 4.42	136 ± 7.23	138 ± 3.21	144 ± 1.23	143 ± 4.02
3	102 ± 4.42	134 ± 7.23	134 ± 4.12	140 ± 5.64	139 ± 2.24
5	104 ± 4.21	137 ± 6.21	138 ± 6.51	133 ± 5.64	135 ± 2.24
7	105 ± 6.64	128 ± 5.21	136 ± 1.23	128 ± 2.32	126 ± 3.54
9	106 ± 0.81	132 ± 0.56	138 ± 0.56	122 ± 1.23	119 ± 6.51
12	104 ± 0.81	138 ± 0.75	134 ± 0.81	116 ± 0.56	111 ± 1.31
14	102 ± 0.81	126 ± 0.75	130 ± 0.81	104 ± 0.56	103 ± 2.52

## 4 Discussion

The extraction method used for MPM produced a good yield 14%w/w. MPM presented similar physicochemical characteristics as reported previously (Ahuja et al., 2011). In this work, MPMLP-NPs formulated by chemical reaction with calcium chloride as a cross-linker in DMSO. Here DMSO used as functional solvent because functional solvent can concomitantly worked as stabilizing agent as well as solvent for reactants. DMSO is a polar, aprotic solvent with moderate dielectric constant that lacks acidic hydrogens leaving it unable to form hydrogen bond with other reagents. Main constituent of MPM are glucuronic acid and xylose (Joseph et al., 2013). NaOH was added in hydrolyzed MPM that lead towards production of sodium salt of glucuronic acid by proton substitution of sodium to COOH group of glucuronic acid as COONa, that serve as reactant site of chemical reaction for cross-linker. When this active gel was added drop wise to calcium chloride/DMSO solution than divalent calcium ions ionically substituted at COONa site and join with another molecule of sodium salt of glucuronic acid that leads towards fabrication of MPMLP-NPs (Duggan et al., 2013).

The FTIR spectra of synthesized nanoparticles presented all bands of LP nanoparticles at 3197.9 cm^−1^, 995.27 cm^−1^, 1008.7 cm^−1^, 1458.18 cm^−1^, 933.5 cm^−1^, 1072.4 cm^−1^, 2954 cm^−1^, 1423.47 cm^−1^ and 1260 cm^−1^ with lower intensities, which is likely due to encapsulation of LP in MPMLP-NPs (Abdoon and Atawy 2021). There were not any subsequent bands were recorded, indicating there was no interaction among LP and the remaining constituents. In DSC analysis, as compared the thermogram of MPMLP-NPs revealed absence of endothermic peak of LP, which might recommend loss of LP crystallinity and the conversion toward the amorphous form in MPMLP-NPs preparation (El-Arabya et al., 2020). MPMLP-NPs formulations showed increased drug content and % yield as with increased quantity of DMSO. This might be due to DMSO utilization increased the cross linking of polysaccharides as reported previously (Petronijevic et al., 2013). The surface of the particles was smooth and as mentioned in literature, the surface of nanoparticles influenced the swelling of nanoparticles and thus release of the drug. For the polymeric nanoparticles to be ideally sustained release, it is important that the surface of the particles should be fairly smooth that will further lead to compliance benefits for the patients (Tummala et al., 2015). The particle size range was between 200 and 300 nm, there has been some evidence that the size of the nanoparticles dried in the hot air oven is usually more as compared to those dried through the freeze-drying method. Thus, the drying methods do have impact on the characteristics and specially the size of the nanoparticles (Rahman et al., 2008). Our results presented polydispersity index between 0.321 and 0.461 that showed an appropriate degree of uniformity and homogeneity among particles as per their size distribution. The more the value of PDI is closer to 0.3–0.4, the uniformity of the particles size is observed to be enhanced (Azevedo et al., 2014). F6 formulation showed zeta potential at −14.9Mv, which is fairly within the range of stability and fulfilled the prerequisites of a stable system as stable nanoparticles are easily dispersible and can enhance the solubility. Negative charge on the nanoparticles were observed that was most possibly due to the carboxyl groups of glucuronic acid and galactomannans present in the mucilage of *Mimosa pudica* seed (Iram et al., 2014). Observing the *in-vitro* dissolution performance of MPMLP-NPs, it was noted that the formulations manifested a spurt of drug release within initial 3 to 4 h and then presented sustained release for 24 h. While commercially, available tablet finished its effect before 12 h. Highest regression correlation coefficient (R^2^) values (0.9940, 0.9992 and 0.9927 for F3, F6 and F9 respectively) were observed in Korsmeyer-Peppas Model that rendered it to be the best-fitted model. The values of diffusional exponent (n) also lie above 1 that indicated the Super Case II Transport mechanism of drug diffusion (Vikas et al., 2011, Khan et al., 2022a, Khan et al., 2022b). During our study, neither toxic effect based on acute toxicological study as mentioned in Fig. 7, Fig. 8 nor in behavioral or sleep pattern based on physical observation seen. No sign of any mortality observed for a time duration of 14 days. No significant difference seen at biochemical and histology level of vital organs that clear the sign of no toxicity linked to polymeric mucilage used. On comparison with the control group, two-way ANOVA applied for statistical analysis and significant difference seen between the groups and days. No significant difference among the diseased, diseased + blank seen which showed that model used developed hypertension that was remained fluctuated in untreated diseased groups until 14th day. But in the treated diseased group with marketed formulation (Qsartan) and MPMLP-NPs (F6), significantly difference was seen on comparison with diseased group (Noreen et al., 2022). The pharmacodynamics studies, from the 9th, 12th and 14th day the diseased + MF and diseased + F6 was statistically same/near to the control group and statistically drop in hypertension was seen from diseased and diseased + blank formulation. Furthermore, a clear difference seen between diseased and diseased plus blank group on comparison with controlled group as shown in Fig. 8. Our results clearly showed that MF(Qsartan) and F6 was effective to show their antihypertensive effect. However, a little bit delayed was seen between the S4 and S5 because of sustained effect of F6 than MF (Qsartan) (Kommana et al., 2020).

## 5 Conclusion

In the present study, MPMLP-NPs prepared for improving the antihypertensive activity by providing the sustained effect as compared to available marketed formulation. The MPMLP-NPs analyzed based on physico-chemical studies by FTIR and DSC. MPM nanoparticles also confirmed for the droplet size, polydispersity index, and zeta potential. *In vitro* drug release studies provide prolong and sustained dissolution rate of prepared MPMLP-NPs (F6) with respect to the commercially available product of LP (MF). Prepared MPMLP-NPs proved better compatibility with sustained effect also. *In vivo* toxicology study also proved the safety of formulation. Furthermore, Prepared MPMLP-NPs (F6) also showed the equivalent antihypertensive activity on comparison with marketed formulation (Qsartan) but with sustained effect. Thus, the formulated MPMLP-NPs useful for antihypertensive treatment with low dose as compare to marketed formulation and can be considered as an alternative.

## Declaration of Competing Interest

The authors declare that they have no known competing financial interests or personal relationships that could have appeared to influence the work reported in this paper.
